# Inhibitory effects of *Azadirachta indica* secondary metabolites formulated cosmetics on some infectious pathogens and oxidative stress radicals

**DOI:** 10.1186/s12906-019-2538-0

**Published:** 2019-06-10

**Authors:** Sunday O. Okoh, Omobola O. Okoh, Anthony I. Okoh

**Affiliations:** 1 SAMRC Microbial Water Quality Monitoring Center, University of Fort Hare, Alice, South Africa; 2 Applied and Environmental Microbiology Research Group (AEMREG), Department of Biochemistry and Microbiology, University of Fort Hare, Private Mail Bag X1314, Alice, Eastern Cape South Africa; 3 Department of Chemical, Environmental and Fibre Technology, FIIRO, Lagos, Nigeria; 4 Department of Pure and Applied Chemistry, University of Fort Hare, Alice, 5700 South Africa

**Keywords:** *Azadirachta indica*, Secondary metabolites, Bactericidal, Radical scavenger, Neem cosmetics

## Abstract

**Background:**

Infectious diseases, particularly those due to multi-drug resistant bacterial strains are almost impossible to cure globally. In this study we investigated the inhibitory effects of *Azadirachta indica* A. Juss secondary metabolites (AISM) formulated soap and cream for management of infectious and oxidative stress-related diseases (OSD).

**Methods:**

The antibacterial, radical scavenging and cytotoxic effects of the neem cosmetics were examined by serial dilution, spectrophotometric and hemolytic techniques respectively, while the AISM in the essential oils (EOs) were elucidated by Gas chromatography-mass spectrometry (GC-MS) and retention index.

**Results:**

The neem cosmetics without AISM exhibited bacteriostatic effects against five reference bacterial strains (*Staphylococcus aureus*, Listeria ivanovii, *Enterobacter cloacae*, Mycobacterium smegmatis, and *Streptococcus uberis*) and two confirmed multi-drug resistant bacterial strains (Vibrio paraheamolyticues, *Escherichia coli* 180) at 0.80 mg/mL. Conversely, at less than 0.50 mg/mL the neem soap produced with AISM demonstrated bactericidal effects against most of these test pathogens linked to infectious diseases. The neem soap containing AISMs displayed noteworthy effects in scavenging radicals associated with OSD at < 1.76 mg/mL. The cosmetics were not toxic to human red blood cells below 0.70 mg/ mL. To our known, the AISM predominantly caryophyllene (30.02%), phytol (14.12%), elemene (13.40%) and linoleic acid (10.5%) exceptional inhibitory effects in neem cosmetics are reported here for the first time.

**Conclusion:**

The study indicates that apart from traditional uses of *A. indica*, the EO contained potent bioactive AISM and feasible as an antimicrobial agent, an alternative to synthetic antioxidant, likewise considered novel in the pharmaceutical, cosmetics industries and as food preservatives.

**Electronic supplementary material:**

The online version of this article (10.1186/s12906-019-2538-0) contains supplementary material, which is available to authorized users.

## Background

The discovery of antibiotics created high hope for management for both infectious and non-communicable diseases, but these hopes have been decreased by the emergence of antibacterial resistant pathogens. Antimicrobial resistance has been listed as one of the top 3 threats to global public health [1]. Bacteria strains including *Escherichia coli*, vancomycin-resistant *Enterococcus faecalis*, Vibrio Spp., and methicillin-resistant *Staphylococcus aureus* (MRSA) are known public threats in hospitals and have become more resistant because of natural selection processes, and over-prescription and misuse of antibiotics [2]. A novel curative agent is desirable which would have to be distinct from current classes of antibiotics to alleviate problems with cross-resistance and co-resistance of microbes [3]. Pharmaceutical industries are unwilling to invest large sums of money into the development of new synthetic antibiotics due to high cost, time-consuming and because bacterial agents rapidly develop resistance no sooner than they were discovered. Studies in recent years have suggested that most secondary metabolites (SM) in essential oil (EO) has better properties and might stand as a substitute against certain pathogenic bacterial strains [3–5].

Most often natural radical scavengers such as catalase, glutathione, peroxidase, superoxide dismutase, and others endogenous antioxidant are overwhelmed by radicals produced including hydroxyl (HO •), lipid peroxyl (LP •), nitric oxide (NO •), superoxide (O2-) from metabolism and ecologically made stressor activities [4]. Consequently, oxidative stress diseases including arthritis, arteriosclerosis, Alzheimer, and cancers, among others grows with cellular injury [6]. Studies have shown that most SM in EO could function as a credible option to man-made antibiotics because they can effectively scavenge radicals and inhibit bacterial and fungus cell growth [7, 8]. Thymol, carvacrol, limonene, caryophyllene, and among others are examples of plant’s SM that possess such bioactive properties [8, 9].

*Azadirachta indica* (Neem tree) is perhaps one of the most useful traditional medicinal plants especially in many tropical and subtropical countries. During the last three decades, apart from the chemistry of the neem essential oil compounds, considerable progress has been achieved on the organic and aqueous extracts biological activity as well as medicinal applications. The solvent extracts were reported to contain bioactive SM including nimbandiol, azadirachtin and nimbinin and natural steroids [10, 11]. Several articles on the usefulness and composition of the seed oil have been published [11–13]. However, there is a dearth of information on the comparative study of the bioactivity of neem cosmetics as well as effects of *A. indica* secondary metabolites (AISM) from the seed oil, leaves and stem-bark EOs on neem products. Such study will unveil the chemistry and broadened the bioactivity knowledge of the plant extracts and amplify it utilizations against infections including ingrown nail, eczema, pimples and skin cancers. We aimed to investigate in vitro the inhibitory effects of soap and cream produced with the neem seeds oil and EOs against some selected infectious pathogens and oxidative stress radicals.

## Methods

### Collection and processing of materials

The chemicals and reagents used included the following: Mueller Hinton agar from Oxford Ltd. (Hampshire, England), Dimethyl sulfoxide (DMSO) from Fluka Chemicals (Buchs, Switzerland). 2, 2-diphenyl-1-picrylhydrazyl (DPPH) were bought from Sigma - Aldrich (St Louis, USA). Reagents used were all analytical grade. The industrial cosmetics chemicals (caustic sodium, sodium silica, glycerol, shea butter, sodium citrate, cetyl alcohol, stearic acid, vitamin E, and propylparaben were bought from industrial soap and cosmetics market Ojota, Lagos, Nigeria. The *A. indica* dried seed (500 g) was bought from National research institute for chemical technology, Zaria, Nigeria. The *A. indica* leaves (200 g) and stem-bark (200 g) were collected in July 2015 at the Digital Institute Bridge, Lagos, Nigeria. A plant taxonomist (Mr. T.K. Odewo) at the Department of Botany, University of Lagos authenticated the three samples (neem seed, leaf, and stem-bark). The three voucher specimens (reference numbers LUH 2009A, LUH2009B, and LUH2009C) were kept in the Lagos University herbarium (LUH). The neem seed oil (fixed oil) was extracted with Federal Institute of industrial research Lagos Nigeria mechanical cold press at room temperature (25.3 ± 2 °C). The EOs were extracted from the air-dried leaves and stem-bark as described in the previous report [14]. The yield of each extract was calculated per gram of the plant material and the EOs were kept in vials at 4 o C until further analysis.

### Analysis of the extracted oils and neem cosmetics produced

The physicochemical properties of the neem oil (NMO) and neem soap were performed using guidelines of AOAC [15] and as described by Fatiany et al. [16]. (a) Acid value: The AV (mg/ KOH/g) of the NMO was evaluated by neutralizing the free acids by KOH; (b) Saponification value (SV): The SV (mg/ KOH/g) was calculated as the quantity of KOH necessary to saponify esters of acids and to neutralize the free acids in one gram of the NMO; (c) Ester index (EI): The EI was quantified as the difference between the SV and the AV. (d) Relative density (RD): The RD was measured with an Anton Paar GmbH dosimeter (DMA35N USA); (e) Refractive index (RI): The neem oil RI was measured at 38 °C with a digital refractometer (ATC95200–009 France). The physicochemical parameters (pH, moisture, total fatty matter, total free caustic alkali, unsaponifiable matter, matter insoluble in ethanol and water) of the neem soaps were carried out as described in Mak-Mensah et al. [17] report. The SM in the leaves EO (LEO) and stem-bark EO (SBEO) were analyzed by gas chromatography-mass spectrometry (GC/MS) and retention index (RI). The instrument used (Hewlett- Packed 5973 mass spectrometer linked with an HP 6890 gas chromatograph) was programmed as in our previous report [14]. Thereafter, the identification each SM was carried out by the conformity of their mass spectra data (MSD) with the reference held in the computer library (Wiley 275, New York). Furthermore, the retention index (RI) of the SM was matched with those in literature, while the total percentage composition of SM in each EO was their peak areas.

### Production of neem cosmetics

The neem soap (NMS) was produced using standard recipe Hui [18] for conventional toilet soap making with the neem seed oil substituted for palm kernel oil (PKO) which provides the triglyceride (fatty acid). The full boiled method was used to produce sample. Briefly, the weighed neem oil and shea butter (Table 1) were warmed at 60 °C. The oil was filtered and poured into a 500 L beaker. Caustic soda solution (10% sodium hydroxide and 20% water) was added to the oil, stirred with a dried wooden stirrer for proper mixing. The mixture was heated, boiled and allowed to become slurry on continuous stirring. On cooling (34 ^o^ C), the slurry was divided into four equal weights, sodium silicate added to each and stirred properly. To the first part (NMS) no neem essential oil was added (as control), in the second part the extracted neem leaves EO was added (NMLEO), for the third part EO from stem-bark was added (NMSBEO), and the fourth contained both NMLEO and NMSBEO (1,1). Each slurry was separately stirred for proper mixing and left in a separate wooden mound to solidify. Thereafter, they were cut into smaller sizes and left at ambient temperature for 48 h prior to analysis.

**Table 1:** Neem soap recipe

Neem oil	Shear butter	Caustic Soda	Sodium silicate	Deionized water	NMS Water^a^	NMLEO	NMSBEO	NMLEO& NMSBEOs^b^	Total
58.75 (% w/w)	4.00	10.00	4.65	21.00	0.40	0.40	0.40	0.40	100%

^a^NMS = negative control

^b^NMLEO & NMSBEO = neem leaves EO and neem stem-bark EO

The neem cream was produced using varnishing base formulation by combining deionized water phase (DWP) and neem seed oil (NSO) as oil phase (OLP) with the use of the emulsifier as described by Hui [17]. Briefly, the OLP was prepared by combining all the waxes (Table 2) and NSO in a microwavable container and heat until they are melted at 82.2 °C. The DWP was made by heating water separately to 75 °C, thereafter citric acid, methylparaben, and propylparaben were dissolved. The mixture then added to the OLP and blended thoroughly. On cooling (32 o C) it was divided into four equal weights in sterilized beakers. Subsequently, the 4 different neem cream samples (NMC, NMCL, NMCSB, and NMCLSB + NMCSB) were produced in a similar model as described four soap samples. They were kept in sterilized bottles at ambient temperature prior to analysis.

**Table 2:** Neem cream recipe

Neem oil	Emulsifier	Glycerol	Vitamin E	Sodium citrate	Propyl paraben	Cetyl alcohol	Stearic Acid	Deionized water	NMC ^a^	Total
8.00 (w/w)	6.00	5.60	1.60	1.00	1.00	1.20	0.80	74.00	1.60	100%

^a^ NMC = neem creams in four different models as in soap (NMC, NMLEO, NMSBEO NMLEO& NMSBEO)

### Antibacterial test

Five reference bacterial strains and two laboratory strains from our laboratory stock culture confirmed to be multi-drug resistant bacteria [19, 20] were used for the antibacterial assay. The reference and laboratory strains bacteria are four Gram-positive*: S. aureus* (NCIB 50080), *M. smegmatis* (ATCC 700084), *L. ivanovii*, (ATCC 19119), *S. uberis* (ATCC700407) and three Gram-negative bacteria: *E. cloacae* (ATCC 13047*), E. coli* 180 and *V. paraheamolyticues* reported to be resistant to sulphamethoxazole, ampicillin, streptomycin, cefuroxime, cephalexin, tetracycline and nalidixic [21] were tested against the neem cosmetics (NC), following CLSI (2014) procedures. The bacterial suspensions were made by inoculating a fresh stock culture of the test bacteria strains into tubes containing 5 mL of sterile Luria Bertani broth and incubated for 24 h at 37 °C. Thereafter, active cultures were grown 24 h in sterile Luria- Bertani broth inoculated into Mueller-Hinton Agar (MHA) incubated for 24 h at 37 °C. After incubation, single colonies were transferred from MHA plates into 4 mL of normal saline solution determined spectrophotometrically at 580 nm as previously reported by Omoruyi et al. [22] and adapted by Igwaran et al. [23] and the dilutions matching with 0.5 Mc-Farland standards were used for the assay.

The modified method of Gullon et al. [24] was used for the determination of MIC and MBC of the neem cosmetics (NC). Under the aseptic condition, two-fold serial dilutions were carried in sterile microcentrifuge tubes in a total volume of 100 μL of Muller Hinton (MH) broth mixed with the NC of various concentrations ranging from 0.0125–0.800 mg/mL. The positive and negative controls were ciprofloxacin and water respectively, thereafter they were incubated at 37 °C for 24 h. The was assay carried in triplicate and tubes with the lowest concentration without visible growth were reported as the minimum inhibitory concentration (MIC). The minimum bactericidal concentration (MBC) for each neem product and the control were determined by streaking out all tubes without visible growth in the MIC technique into fresh nutrient agar plates. Then, at 37 °C, the culture was incubated for 24 h. The lowest concentration of the cosmetics and ciprofloxacin that didn’t on the solid medium yield any growth after 24 h [25] was recorded as the MBC.

### Antioxidant test

Three antioxidant assays (DPPH, nitric oxide, and lipid peroxyl) were used to assess the radical scavenging property of neem cosmetics. The DPPH procedures of Liyana-Pathirana et al. [26] as described in the previous report [14] was followed to evaluate the neem cosmetics (NC) inhibitory potentials against DPPH radical in DMSO as diluent. The test was performed in triplicate and the capacity of NC to scavenge DPPH • to a neutral molecule (DPPH-H) was determined as % scavenging effects with the eq. (1) below.

1 $$ \%\mathrm{scavenging}\ \mathrm{of}\ \mathrm{DPPH}\bullet \mathrm{by}\ \mathrm{NC}\ \mathrm{or}\ \mathrm{RCs}=\left\{\left(\mathrm{Abs}\ \mathrm{cl}-\mathrm{Abs}\ \mathrm{sp}\right)\right\}/\left(\mathrm{Abs}\ \mathrm{cl}\right)\times 100 $$

Where Abs cl is the absorbance of the DPPH radical + DMSO; Abs sp. is the absorbance of DPPH radical + NC or reference compounds (RCs).

The antioxidant potency of each NC and reference compound against the lipid peroxyl radical (LP •) were determined by the thiobarbituric acid assay as described in the previous report [14] with a known lipid-rich source (egg yolk). Subsequently, at 532 nm the absorbance of the solution was read after aspirating the upper organic layer. The scavenging efficacy of each NC and RCs against lipid peroxyl at increasing concentrations (0.025–0.50 mg/mL) was assessed with the eq. (1) above.

The sodium nitroprusside nitric oxide radical (NO•) induced protocol as described by Makhija et al. [27] was used to determine the NO• scavenging potential of the NC. At physiological pH 7.2, sodium nitroprusside nitric oxide decomposed generating NO• which react in the presence of O2 and resultant stable nitrite and nitrate is measured in Griess solution. Briefly, 1.0 mL of the NC at double fold concentrations (0.025–0.5 mg/mL prepared in DMSO) was added to 1.0 mL (10 mM) of sodium nitroprusside solution. The solution was incubated for 110 min at ambient temperature. Then followed the color development by adding 1.0 mL of the aliquot to Griess solution (1.0% N-naphthyl-ethylene diamine hydrochloride in 2.0% orthophosphoric acid and 1.0%, sulphanilamide). Thereafter, the absorbance of the color developed was then measured spectrophotometrically at 546 nm against the reagent blank. The scavenging effect (%) was then obtained using equation (a) described in DPPH radical test. The assay was carried out in parallel triplicate and the mean value calculated. The IC50 (mg/ mL) was obtained from the standard curve for each neem product and positive control in the three protocols. The lower the IC50 value the higher the antioxidant capacity.

### Cytotoxicity assay

The in vitro hemolytic technique as illustrated by Helander et al. [28] was followed to assess the cytotoxicity of the NC with few modifications. Hemolytic test investigates hemoglobin discharge in the plasma (indicating red blood cell [RBC] lysis) due to contact with the test sample. Human RBC from one of our projects involving human blood was used in preparing blood agar plates (BAP). Wells were bored on the BAP at 0.10–0.70 mg / mL prepared in deionized water. Then, into each well 25 μL of the neem product was poured while BAP and well with only deionized water was used negative control. Thereafter, at 37 °C, the plates incubated for 24 h. The presence of hemolytic activity was examined on wells and the assay was carried in triplicate.

### Data analysis and calculations

The significant difference between the neem product and controls were carried out by SPSS15.0 for windows (Institution registration No for IBM-SPSS/OLRAC SPS 2012/1786646/07). All protocols results were expressed as means ± S. D of duplicate in cytotoxicity assay while the antibacterial, antioxidant and physicochemical tests were performed in triplicate. Regression equation produced from the standard curve for each sample in the antioxidant assay was used to calculate the IC50 value for each NC and controls. T-Test correlation analysis was used to test significant differences between the % radical scavenging and concentrations. The confidence level at *P* < 0.05 was considered significant.

## Results

### Inhibitory effects of cosmetics on pathogens

The inhibitory effects (supporting document 1) of the neem cosmetics produced with and without AISM on test pathogens are as presented in Tables 3 and 4. The antibacterial activities of the neem soaps produced with AISM (NMLS, NMSBS, and NMLSBS) were significantly different (*p* < 0.05) from those without AISM (NMS) with MIC values ranging between 0.0125 to 0.400 mg/mL and 0.400 to 0.800 mg/mL respectively against the seven test pathogens. The NMS demonstrated a bacteriostatic effect on all test bacterial strains at 0.800 mg/mL, while the neem soap produced with AISM from the leaf EO (NMLS) was bactericidal against *E. cloacae, L. ivanovii, S. aureus*, and *V. paraheamolyticues* at 0.400, 0.400, 0.800 and 0.800 mg/mL respectively. Unlike the NMS and NMLS, the neem soap made with AISM from the stem-bark EO (NMSBS) was bactericidal against all the test pathogens with MBC ranging between 0.100–0.800 mg/mL. The neem soap prepared using AISMs from both leaf and stem-bark EOs (NMLSBS) exhibited the highest antibacterial effects with MIC and MBC values of 0.0125 and 0.025 mg/mL against *L. ivanovii*, and 0.025 and 0.200 mg/mL against *E. cloacae*. Interestingly the NMLSBS and positive control (ciprofloxacin) had same MIC value against *L. ivanovii,* while the MBC (0.025 mg/mL) was significantly better than control drug [ciprofloxacin (0.05 mg/mL)] as shown in Table 3.

**Table 3:** Neem soap minimum inhibitory and minimum bactericidal concentrations (MIC and MBC)

Test Pathogens	Neem Soap								Controls		
	NMS		NMLS ^a^		NMSBS ^b^		NMLSBS ^c^		Ciprofloxacin ^d^		Water ^e^
	MIC mg /mL	MBC mg / mL	MIC mg/mL	MBC mg /mL	MIC mg /mL	MBC mg /mL	MIC mg /mL	MBC mg/mL	MIC mg/mL	MBC mg/mL	MIC 500 μL
*L. ivanovii*	0.400 ± 0.03	0.800 ± 0.01 VG	0.200 ± 0.03	0.400 ± 0.01 NG	0.200 ± 0.02	0.400 ± 0.01 NG	0.012 ± 0.002	0.025 ± 0.001 NG	0.012 ± 0.001	0.05 ± 0.01 NG	VG
*S. aureus*	0.800 ± 0.02	0.800 ± 0.10 VG	0.400 ± 0.04	0.800 ± 0.01 NG	0.400 ± 0.04	0.400 ± 0.03 NG	0.200 ± 0.03	0.200 ± 0.02 NG	0.050 ± 0.001	0.050 ± 0.04 NG	VG
*M.smegmatis*	0.400 ± 0.04	0.800 ± 0.04 VG	0.400 ± 0.12	0.400 ± 0.03 VG	0.400 ± 0.04	0.400 ± 0.11 NG	0.200 ± 0.04	0.400 ± 0.02 NG	0.050 ± 0.02	0.100 ± 0.02 NG	V G
E. coli 180*	0.800 ± 0.03	0.800 ± 0.03 VG	0.800 ± 0.11	0.800 ± 0.04 VG	0.400 ± 0.02	0.800 ± 0.03 NG	0.400 ± 0.01	0.800 ± 0.01 NG	0.050 ± 0.01	0.050 ± 0.02 NG	V G
V.paraheamolyticues *	0.400 ± 0.01	0.800 ± 0.03 VG	0.400 ± 0.03	0.800 ± 0.03 NG	0.400 ± 0.02	0.800 ± 0.03 NG	0.200 ± 0.02	0.400 ± 0.01 NG	0.050 ± 0.01	0.050 ± 0.01NG	V G
*E. cloacae*	0.400 ± 0. 01	0.800 ± 0.04 VG	0.200 ± 0.01	0.400 ± 0.02 NG	0.025 ± 0.020	0.100 ± 0.010 NG	0.025 ± 0.020	0.200 ± 0.010 NG	0.012 ± 0.010	0.012 ± 0.001 NG	V G
*S. uberis*	0.800 ± 0.02	0.800 ± 0.03 VG	0.40 ± 0.01	0.800 ± 0.04 VG	0.40 ± 0.03	0.800 ± 0.03 NG	0.40 ± 0.00	0.800 ± 0.01 NG	0.050 ± 0.01	0.050 ± 0.01 NG	ND

*NMS=* neem soap without neem essential oil

^a^NMLS = neem soap with leaf EO

^b^NMSBS = neem soap with neem stem-bark EO

^c^NMLSBS = neem soap with the leaf and stem-bark EO

^d^ciprofloxacin = positive control

^e^Water = negative control

* confirmed bacterial resistant strains, VG = Visible growth (bacteriostatic), NG = No visible growth (bactericidal) on solid agar plate, ND = Not determined

**Table 4:** Neem cream minimum inhibitory and minimum bactericidal concentrations (MIC & MBC)

Test Pathogens	Neem cream								Controls		
	NMC		NMLC ^a^		NMSBC ^b^		NMLSBC ^c^		Ciprofloxacin ^d^		Water ^e^
	MIC mg /mL	MBC mg / mL	MIC mg/mL	MBC mg /mL	MIC mg /mL	MBC mg /mL	MIC mg /mL	MBC mg/mL	MIC mg/mL	MBC mg / mL	MIC (500 μL)
*L. ivanovii*	0.800 ± 0.03	0.800 ± 0.01 VG	0.400 ± 0.02	0.800 ± 0.01 NG	0.400 ± 0.02	0.800 ± 0.01 NG	0.400 ± 0.02	0.400 ± 0.02 NG	0.012 ± 0.001	0.050 ± 0.01 NG	VG
*S. aureus*	0.800 ± 0.02	0.800 ± 0.03 VG	0.800 ± 0.04	0.800 ± 0.02 VG	0.800 ± 0.03	0.800 ± 0.03 NG	0.400 ± 0.03	0.800 ± 0.02 NG	0.050 ± 0.001	0.050 ± 0.04 NG	V G
*M. smegmatis*	0.800 ± 0.01	0.800 ± 0.02 VG	0.400 ± 0.02	0.800 ± 0.03 NG	0.800 ± 0.02	0.800 ± 0.01 NG	0.400 ± 0.04	0.800 ± 0.02 NG	0.05 ± 0.02	0.100 ± 0.02 NG	V G
E. coli 180*	0.800 ± 0.03	0.800 ± 0.03 VG	0.800 ± 0.01	0.800 ± 0.02 VG	0.800 ± 0.01	0.800 ± 0.01 VG	0.800 ± 0.02	0.800 ± 0.04 VG	0.050 ± 0.01	0.050 ± 0.02 NG	V G
*V.paraheam-olyticues* ^*^	0.800 ± 0.02	0.800 ± 0.03 VG	0.800 ± 0.01	0.800 ± 0.00 VG	0.800 ± 0.11	0.800 ± 0.03 VG	0.800 ± 0.04	0.800 ± 0.03 VG	0.050 ± 0.01	0.050 ± 0.03 NG	VG
*E. cloacae*	0.800 ± 0. 02	0.800 ± 0.04 NG	0.800 ± 0.12	0.800 ± 0.03 NG	0.400 ± 0. 02	0.800 ± 0.01 NG	0.400 ± 0.02	0.800 ± 0.04 NG	0.012 ± 0.001	0.012 ± 0.00 NG	ND
*S. uberis*	0.800 ± 0.02	0.800 ± 0.01 VG	0.800 ± 0.01	0.800 ± 0.03 VG	0.800 ± 0.03	0.800 ± 0.02 NG	0.400 ± 0.01	0.800 ± 0.04 NG	0.050 ± 0.01	0.050 ± 0.04 NG	ND

*NMS* neem cream without neem EO

^a^ NMLC = neem cream with leaf EO, NMSBC = neem cream with stem-bark neem EO

^c^NMLSBC = neem soap with leaf EO and stem-bark EO

^d^ amphotericin B = positive control

^e^Water = negative control, *P* < 0.05

* confirmed bacterial resistant strains

The four neem creams were generally less active against the seven pathogens compared to the neem soaps. Like the neem soaps, the neem creams produced with the AISM stem-bark and from both leaf and stem-bark EO (NMLSBC) had better effects against the pathogens (Table 4). However, at 0.800 mg/mL on *E. cloacae*, the four creams exhibited a similar effect, while NMLSBC displayed a superior MIC value (0.400 mg/mL). The NMLSBC displayed higher inhibitory effects than other neem cream products with lower MBC values against the five test pathogens, however, its effect was lower than that of positive control drug (Table 4).

### Radicals scavenging effects of neem cosmetics

**Fig. 1:**
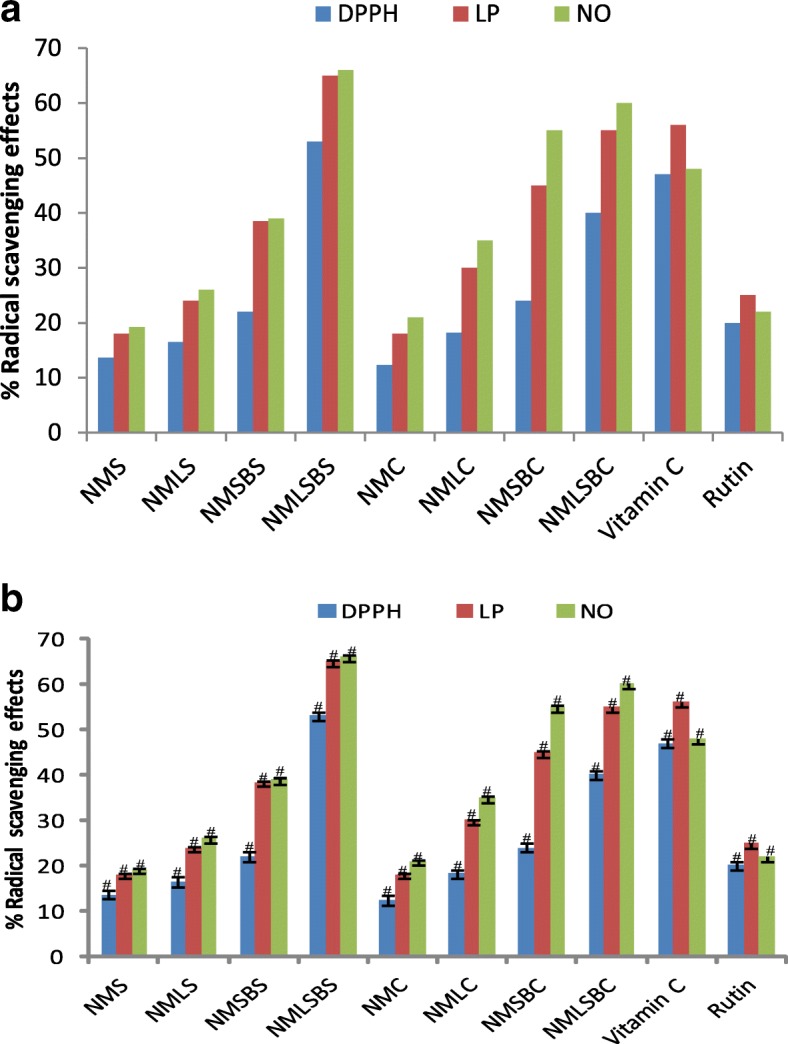
**a** Radical scavenging effects of neem cosmetics with and without secondary metabolites and reference compounds at 0.10 mg/mL. **b** Radical scavenging effects of neem cosmetics with and without secondary metabolites and reference compounds at 0.10 mg/mL (# = significantly different, s = not significantly different *p* < 0.05)

**Fig. 2:**
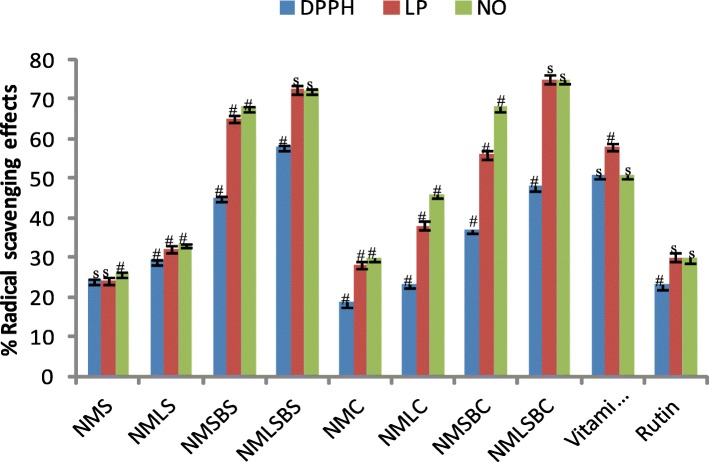
Radical scavenging effects of neem cosmetics with and without secondary metabolites and reference compounds at 0.20 mg/mL (# = significantly different, s = not significantly different *p* < 0.05)

**Fig. 3:**
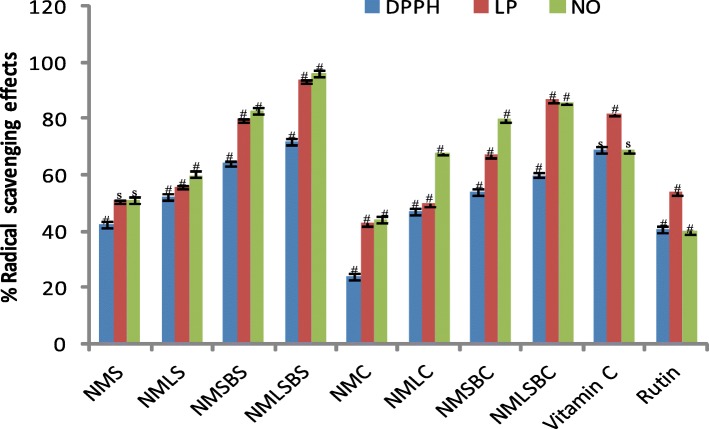
Radical scavenging effects of neem cosmetics with and without secondary metabolites and reference compounds at 0.50 mg/mL (# = significantly different, s = not significantly different *p* < 0.05

The effects of AISM from leaf and stem-bark EOs on the radical scavenging property of neem soaps (NMLS, NSBS, and NMLSBS), creams (NMC, NMLC, NMSBC and NMLSBC) as well as neem cosmetics (NMS and NMC) produced devoid of AISM were examined on three different radicals (DPPH •, LP •, and NO •). The radical scavenging effects of neem soaps and positive control (ascorbic acid and rutin) on the radicals were concentration dependent (Figs. 1, 2 and 3). At low concentration, Table 5 (0.10 mg/ mL) the neem soaps except for NMSBS scavenging effects on the three (DPPH, LP, and NO) radicals were significantly different # (*p* < 0.05), while the effect of NMSBS against LP •, and NO • were similar (s). At a higher dose (0.20 mg/ mL) all test neem soap samples scavenged the three radicals differently (Fig. 2). Similarly, at the highest concentration (0.5 mg/mL) the neem soaps excluding the soap without AISM scavenging effects were significantly different # (< 0.05) as showed in Fig. 3.

**Table 5:** Neem cosmetics scavenging effects on different radicals at 0.10 mg/mL

NCs at 0.10 mg/mL	DPPH	LP	NO
NMS	13.6	18.0	19.2
NMLS	16.5	24.0	26.0
NMSBS	22.0	38.45	39.0
NMLSBS	53.0	65.0	36.0
NMC	12.3	18.0	21.0
NMLC	18.2	30.0	35.0
NMSBC	24.0	45.0	55.0
NMLSBC	40.0	55.0	60.0
Vitamin C	47.0	56.0	48.0
Rutin	20.0	25.0	22.0

*NMS &NMC* neem soap and cream without neem EOs, *NMLS& NMLC* neem soap and cream containing leaf EO, *NMSBS & NMSBC* neem soap and cream containing neem stem bark EO, *NMLSBS & NMLSBC* neem soap and cream containing both leaf and stem bark EOs

A substance that offers one or more hydrogen atoms to a radical converting the latter a non- radical molecule is referred to as a radical scavenger. This activity is shown as the color of DPPH • turns to yellow from purple (DPPH • solution) of the investigated substance because of formation of a neutral molecule of DPPH-H upon donation of H atom from the substance [29]. The strength of the examined radical scavenger is the reduction of UV absorbance at 517 nm. However, DPPH technique doesn’t distinguish radical type but an overall radical scavenging potency of a substance [14]. We therefore quantitatively tested presumed and précised inhibitory efficiency of each neem product by employing two different specific types of radicals (nitric oxide and lipid peroxyl) associated with skin inflammation, blood diseases, and other oxidative stress-related ailments. Evaluating the IC_50_ values for the different soaps and creams, regression equations from the standard curves generated for each radical was used while t-test analysis was employed for significantly different of % radical scavenging effects against the concentrations (Figs. 1, 2 and 3). The neem cosmetics made without the AISM exhibited mild antioxidant capacity against nitric oxide, lipid peroxyl and DPPH radicals with IC50 values ranging between 4.78–6.50 and 6.10–10.42 mg/mL for the soaps and creams respectively. The cosmetics produced using AISM from the neem leaf and stem-bark EOs demonstrated the highest antioxidant efficacy against three distinct radicals with IC_50_ values 1.65–2.60 mg/mL for the neem soap (NMLSBS). Interestingly, the antioxidant capacities of NMLSBS and NMLSBC were remarkably better than the two positive control drugs (Table 6) against LP and NO radicals associated with OSD.

**Table 6:** Antioxidant capacity of neem cosmetics and reference compounds (mg / mL)

Neem samples sand reference compounds		Activity	
	DPPH ^•^	LP ^•^	NO ^•^
NMS (IC_50_)	6.50 ± 0.12	5.81 ± 0.03	4.78 ± 0.12
NMLS (IC_50_)	5.50 ± 0.01	5.11 ± 0.02	4.06 ± 0.02
NMSBS (IC_50_)	4.30 ± 0.10	3.26 ± 0.01	3.06 ± 0.03
NMLSBS (IC_50_)	2.60 ± 0.02	1.75 ± 0.03	1.65 ± 0.01
NMC (IC_50_)	10.42 ± 0.12	6.23 ± 0.03	6.10 ± 0.02
NMLC (IC_50_)	5.48 ± 0.01	5.21 ± 0.02	3.94 ± 0.02
NMSBC (IC_50_)	5.10 ± 0.10	3.41 ± 0.01	2.49 ± 0.03
NMLSBC (IC_50_)	4.05 ± 0.02	2. 23 ± 0.03	1.50 ± 0.01
Rutin (IC_50_) ^*^	5.74 ± 0.02	5.26 ± 0.11	4.80 ± 0.04
Vitamin C (IC_50_) ^*^	3.18 ± 0.01	2.28 ± 0.03	2.16 ± 0.01

*NMS &NMC* neem soap and cream without neem EOs, *NMLS& NMLC* neem soap and cream containing leaf EO, *NMSBS & NMSBC* neem soap and cream containing neem stem bark EO, *NMLSBS & NMLSBC* neem soap and cream containing both leaf and stem bark EOs

^*^positive control drugs, ± SD, *n* = 3. P < 0.05 was considered significant

### Physicochemical of properties of neem seed oil, essential oils, and neem cosmetics

The physicochemical properties (PCP) of the crude neem seed oil (NSO) and essential oils (EOs) extracted by mechanical cold press and hydrodistillation from the aerial parts respectively are as presented in Table 7. Except a lower iodine value (IV) of 77.47% obtained, the saponification, refractive index, and acid values of the NSO were similar to previous reports of neem seed oil [17, 30, 31] and some vegetable oils used for cosmetics making, especially soap [32]. However, the IV in this present study was higher than the result obtained (63.81%) for neem seed oil grown in Sudan [31, 33]. From Table 7, it can be noticed that the relative density (RD) of the NSO and the two EOs is less than 1. However, the RD of the crude NSO (0.921) was significantly lower (*p* < 0.05) than that reported for neem seed oil (0.939) by Akpan, [34] and higher (0.905) than that obtained in India [35]. The RD values for EO from leaves (0.0902) and stem-bark (0.904) was significantly different p < 0.05 from the crude NSO. The acid values of the EOs were found to be greater than NSO. Conversely, the ester, refractive indices and saponification values of the NSO were greater than that of leaf and stem-bark EOs.

**Table 7:** Physicochemical of properties of neem oil and essential oil

Physicochemical Properties	Neem oil^a^	Leaf EO^b^	Stem-bark EO
Physical state	viscous liquid	light viscous liquid	light viscous liquid
Colour	brownish	light yellow	colourless
Odour	garlic repulsive	repulsive	repulsive
Relative density	0.921 ± 0.014	0.904 ± 0.113	0.902 ± 0.103
Acid value (1 mg of KOH/g of oil)	15.840 ± 0.0021	17.191 ± 0.004	19.630 ± 0.001
Ester index	172.201 ± 0.012	74.930 ± 4.311	53. 150 ± 1.866
Refractive index at 38 °C	1.4667 ± 0.011	1.4383 ± 0.031	1.4372 ± 0.201
Saponification value	187.620 ± 0.031	109.312 ± 0.021	72.780 ± 0.011
Iodine value (%) (g of I2/100 g of oil)	77.470 ± 0.011	ND	ND

^a^oil extracted from the neem seed by mechanical press

^b^essential oil extracted from the leaves and stem-bark by hydrodistillation, ND = not determined

The physicochemical properties of neem soaps produced with and without the AISM are as presented in Table 8. The chemical properties of neem soaps were within the control limits of South Africa specifications for antibacterial toilet soap, Kenya standard for commercial toilet soap and standard Organisation of Nigeria (SON) for toilet soap [36–38], except the free caustic alkali (0.32% as Na_2_O) for the neem soap (NMS) produced without AISM. Nevertheless, the total fatty matter (77.30–80.14%) for the four neem soaps and other physicochemical parameters were better than the specifications of the SON for toilet soap and similar to neem soap produced elsewhere [17].

**Table 8:** Physicochemical properties of neem soap and SON specifications for toilet soap

Physicochemical Properties	NMS	NMLS	NMSBS	NMLSBS	SON* Control
Colour	milky	milky	milky	milky	–
pH. (10%)	9.60 ± 0.03	9.40 ± 0.02	9.36 ± 0.03	9.34 ± 0.01	9–11 max ^a^
% Moisture content	14.45 ± 0.02	12.50 ± 0.00	13.42 ± 0.03	13.36 ± 0.02	13–20 max
%Total fatty matter	77.30 ± 0.04	77.68 ± 0.00	80.02 ± 0.01	80.14 ± 0.02	76.50 mini ^b^
Total free caustic alkali as Na_2_O	0.32 ± 0.03	0.14 ± 0.01	0.12 ± 0.02	0.13 ± 0.00	0.20 max
% Matter insoluble in ethanol	0.40 ± 0.01	0.03 ± 0.04	0.22 ± 0.00	0.20 ± 0.02	2.00 max
% Matter insoluble in water	0.35 ± 0.00	0.33 ± 0.02	0.26 ± 0.01	0.23 ± 0.04	0.50 max
% Unsaponified Matter	0.23 ± 0.01	0.13 ± 0.02	0.04 ± 0.01	0.22 ± 0.01	0.50 max
Texture	Hard	very hard	very hard	very hard	–
Leathering	Good	excellent	excellent	excellent	–

Standard organisation of Nigeria specifications for toilet soap ± SD, *n* = 3. *P* < 0.05 was considered significant. NMS = neem soap without neem EO, ^a^ NMLS = neem soap with leaf EO, NMLSBS = neem soap with leaf and stem bark EOs. a = maximum limit, b = minimum limit

### Cytotoxicity of the neem cosmetics

The cytotoxicity effects on neem cosmetics are as presented in Table 9. DMSO in blood agar without the cosmetics was used as negative control. The neem soap produced using AISM from both the leaf and stem – bark (NMLSBS) exhibited low toxic (slight lysis) effect at 0.350 mg/ mL, while none of the cosmetics displayed acute toxic effect at less than 0.70 mg/mL on human RBC except NMLSBS.

**Table 9:** Cytotoxic effect of neem cosmetics on human blood RBC

Sample	Concentration (mg/ mL)						
	0.10	0.15	0.20	0.25	0.30	0.35	0.70
NMS	–	–	–	–	–	–	–
NMLS	–	–	–	–	–	–	+
NMSBS	–	–	–	–	–	–	++
NMLSBS	–	–	–	–	–	+	+++
NMC	–	–	–	–	–	–	–
NMLC	–	–	–	–	–	–	–
NMSBC	–	–	–	–	–	–	–
NMLSBC	–	–	–	–	–	–	+
Water^a^	–	–	–	–	–	–	–

^a^water in blood agar without cosmetics; − = No lysis (no toxic effect); + = slight lysis (low toxic effect); ++ = mild lysis (moderate toxic effect); +++ = acute lysis (sever toxic effect

### Secondary metabolites in the neem cosmetics

The chemical profiles of secondary metabolites (SM) isolated from *A. indica* leaf and stem-bark incorporated unto the recipes of neem cosmetics are as presented in Table 10. In this current study terpenes and oxygenated terpenes (terpenoids) that were the chief SM, efficiently boosted the antibacterial and antioxidant properties of neem cosmetics. Fifteen SMs were found in the LEO, while the SBEO contained 19 which represents 98.91 and 99.45% of the total SM content respectively (Table 10). In the LEO, α-caryophyllene, γ-elemene and hexadecane were the major sesquiterpenes, accounting for 44.30% of the overall SM content, while the total monoterpenes, monoterpenoids and sesquiterpenoids SM were 22.70, 10.50 and 4.51% respectively. Conversely, in the SBEO there were fewer sesquiterpenes SM (24.21%) compared to the SM in the LEO. However, there were more sesquiterpenoids (23.70%) and monoterpenoids (19.80%) SM in SBEO. The presence of bioactive SMs including a diterpenoid (phytol 9.21%), curcumene (7.8%), camphene (3.0%), ledol (2.4%) and tetracosane (1.32%) in SBEO absent in LEO remarkably distinguished the chemical profile of the stem-bark EO from the leaf (Table 10). Furthermore, the yields of linalool oxide (15.2%), terpinene (13.7%), hexadecenoic acid (10.2%) and polyunsaturated linoleic acid (5.40%) were significantly higher in SBEO. This may corroborate the superior bioactive and physiochemical properties observed in neem stem-bark cosmetics products (NMSBEOS, NMLEOS-NMSBEOS, NMSBEOC, NMLEOC-NMSBEOC) in this study.

**Table 10:** Chemical profiles of secondary metabolites in *A. indica* essential oils

S/N	SM ^a^	KI ^b^	%composition		MI ^c^	QA ^d^	MS data ^e^	MF^*^
			LEO	SBEO				
1	α-Pinene	927	4.10	0.50	MSD, KI	99	79, 93,136	C_10_H_16_
2	Camphene	944	–	3.00	MSD, KI	97	93, 69,136	C_10_H_16_
3	Myrcene	970	5.60	t	MSD, KI	99	41, 93, 69	C_10_H_16_
4	Limonene	1031	10.4	5.50	MSD, KI	98	68, 93, 79	C_10_H_16_
5	Terpinene	1063	2.70	13.70	MSD, KI	96	77, 93, 121	C_10_H_16_
6	Linalool oxide	1068	6.40	15.20	MSD, KI	99	91, 41, 43	C_10_H_16_
7	Linalool	1089	4.10	4.60	MSD, KI	95	44, 71,113	C_10_H_18_O
8	β-Curcumene	1120	–	7.81	MSD, KI	97	109,161, 43	C_15_H_22_
9	α-Cedrene	1129	2.20	t	MSD, KI	91	93, 41, 204	C_15_H_24_
10	α –Cubebene	1369	1.60	3.00	MSD, KI	97	43, 68, 161	C_15_H_24_
11	γ-Elemene	1385	10.20	5.10	MSD, KI	99	43, 68, 204	C_15_H_24_
12	α- Caryophyllene	1415	30.30	8.30	MSD, KI	98	93, 41, 79	C_15_H_24_
13	Caryophyllene oxide	1545	2.40	5.10	MSD, KI	98	41, 79, 111	C_15_H_24_O
14	Ledol	1560	–	2.40	MSD, KI	96	93, 79, 133	C_15_H_26_O
15	Hexadecanoic acid	1968	5.40	10.20	MSD, KI	96	43, 60, 73	C_16_H_32_O_2_
16	Heptadecane	2030	2.40	2.30	MSD, KI	95	43, 60, 256	C_17_H_36_
17	Linoleic acid	2039	2.11	5.40	MSD, KI	98	73,265,279	C_18_H_32_O
18	Phytol	2045	–	9.12	MSD, KI	99	41,71, 123	C_20_H_40_O
19	Tetracosane	2105	–	1.32	MSD, KI	98	73, 43, 129	C_24_H_50_
% Total			98.91	99.45				
% Yield			0.72	0.58				

^a^Secondary metabolites listed in order of elution from HB-5 column

^b^Retention indices relative to C_9_ - C_25_ n-alkanes on column

^c^methods of identification (mass spectra data & KI = retention index)

^d^Quality assurance of GC/MS library

^e^mass spectra data

^*^molecular formula t < 0.03%, LEO = leaves essential oil, SBEO = stem bark essential

## Discussion

Biochemical investigations of inhibitory effects on pathogens and radicals of two different neem extracts (neem seed oil and EO) revealed higher bioactive and physicochemical properties of NC formulated with SM from the EO. Characterization of SM in the leaf and stem EOs integrated into the recipe of NC shown more terpenoids including phytol 18, one sesquiterpenoid (ledol 14), a monoterpene (camphene 2) and sesquiterpene (curcumene 8) in the stem-bark (SBEO). Moreover, the content of bioactive linoleic acid (17), hexadecenoic acid (15), caryophyllene oxide (12), linalool oxide (6) and terpinene (5) were more in SBEO. Accordingly, the NC made incorporating SMs from SBEO and LEO had the highest activity followed by NC produced with the SM from the SBEO, while NC produced with only crude neem seed oil (NSO) displayed the least activity. To the best of our knowledge, this enhanced bioactivity of neem cosmetics formulated from the leaves and stem bark of *A. indica* SMs are reported here for the first time in the genus *Azadirachta*. The neem cosmetics produced with AISM were more active against Gram-positive bacterial than the test Gram-negative bacterial strains. This may be linked to net repulsion of the complex outer membrane in Gram-negative bacterial, reported in previous studies to contain hydrophilic lipopolysaccharide which exhibits higher tolerance toward hydrophobic terpenes and oxygenated terpenes [4, 39]. Another probable cause of resistance is the presence of multi-drug resistant sites that promote the synthesis and secretion of amphipathic toxins [40].

Phytol one of the major SM in the SBEO was reported [41] to demonstrate good antioxidant effects in vivo and has a high capacity to quench hydroxyl and nitric oxide radicals as well as prevent the formation of lipid peroxides analyzed via thiobarbituric acid reactive substances (TBARS). Phytol may have possibly reacted with nitric oxide, lipid, DPPH radicals, and the seven test bacterial strains via various mechanisms suggested by Mortein et al. and Foti and Amorati [4, 42]. In addition, terpinene a major SM we found in SBEO of *A. indica* is known to inhibit low-density lipoprotein oxidation even in the early phase [4]. Other bioactive SMs in EO profile including camphene [43], pinene [44] and linoleic acid [45] may have heightened the anti-oxidation and inhibition effects of the NC on radicals and the test pathogens.

**Fig. 4:**
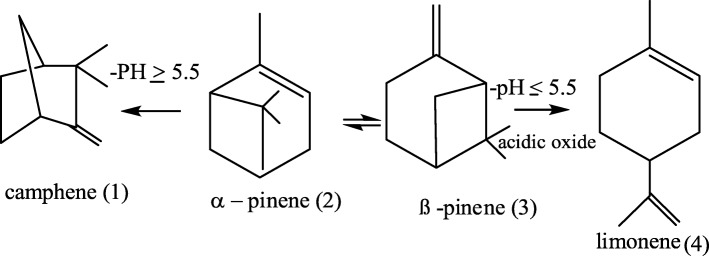
Chemical transformation of some secondary metabolites

Based on their chemical structure, bioactive SMs are primarily terpenoids, terpenes, phenylpropenes and transformed products that originate either from terpenes, and unsaturated fatty acids [46] prominent in the EOs of *A. indica*, especially in the stem-bark (Table 10). This indicates the possibility of transformation of SM in the stem-bark and the entire parts of *A. indica*. A study by Gomes et al. [47] on the variation of SM in EO plants parts, revealed that limonene and caryophyllene are the most SMs that contribute frequently for the monoterpenes and sesquiterpenes biotransformation in EOs. This report supports the results we obtained as shown in the varied quantities of limonene and caryophyllene we found in the EOs; which might have transformed due to stress factors including temperature and pH. Study on SMs by Corma et al. [48] shown that these terpenes are pH sensitive and are bio-transformed via isomerization (Fig. 4).

The comparison of inhibitory effects of NC and commercial antiradicals as well as standard antibiotics is a demonstrable strong indication of NC produced as an antibacterial and antioxidant agent especially the neem products formulated from stem bark SM. The ability of NMLSBS and NMLSBC to effectively reduced 3 types of free radicals to non-radical molecules and display significant bioactivity against five reference bacterial strains and two laboratory-confirmed multi-drug resistant bacterial strain is noteworthy. Therefore, the increased bioactive and physiochemical properties displayed by the SM in the neem cosmetics (NMSBS and NMSBC) indicates that the stem-bark EO will be feasible as a fresh potent source in the hunt for principal agent for the management of oxidative stress-related ailments such as skin cancers [49], scabies, impetigo, and other related contagious skin diseases in this era of emerging multidrug resistance microorganisms.

None of the neem cosmetics displayed hemolytic effects on human red blood cells (HRBC) below 0.68 mg/ mL. Mild cytotoxic effect was observed in neem soap formulated with combined stem-bark and leaf SMs at > 0.69 mg/ mL. This may be attributed to the frail nature of HRBC since there has never been any report on human toxicity after using a decoction of *A. indica* leaves and stem-bark in tropical and subtropical countries.

## Conclusion

This present study indicates that besides the known uses neem tree, the neem cosmetics integrated with the stem-bark and leaf essential oil has robust bioactive secondary metabolites; therefore, the EO could be a novel applicant as new antimicrobial agent in this current era of rising multidrug-resistant bacterial strains, likewise as an alternative to synthetic antioxidant and effective bioactive chemical in pharmaceutical and cosmetics industries.

## Additional file

Additional file 1: Screening of the products against 7 Bacterial strains at different concentrations. (DOCX 4399 kb)

## Data Availability

All data and materials used in the study are in the manuscript also in the supplementary information files attached Additional file 1.

## Abbreviations

AISM: *Azadirachta indica* secondary metabolites
DMSO: Dimethyl sulphur oxide
DPPH•: 2,2-diphenyl-1-picrylhydrazyl radical
EOs: Essential oils
GC-MS: Gas chromatography-mass spectrometry
IC50: Inhibitory concentration at 50%
LP•: Lipid peroxide radical
MBC: Minimum bactericidal concentration
MIC: Minimum inhibition concentration
NC: Neem cosmetics
NMLS& NMLC: Neem soap and cream containing leaf EO
NMLSBS & NMLSBC: Neem soap and cream containing both leaf and stem bark EOs
NMO: Neem oil
NMS &NMC: Neem soap and cream without neem EOs
NMSBS & NMSBC: Neem soap and cream containing neem stem bark EO
NO•: Nitric oxide radical
OSD: Oxidative stress-related diseases
