# Supplementary material for: Inhibitory effects of Azadirachta indica secondary metabolites formulated cosmetics on some infectious pathogens and oxidative stress radicals
Source: BMC Complement Altern Med. 2019 Jun 10;19:123. doi: 10.1186/s12906-019-2538-0 (PMC6558881; doi:10.1186/s12906-019-2538-0)
Supplement: Supplementary file 1 — Screening of the products against 7 Bacterial strains at different concentrations. (DOCX 4399 kb) [file 12906_2019_2538_MOESM1_ESM.docx]

**SUPPORTING DOCUMENTS**

**
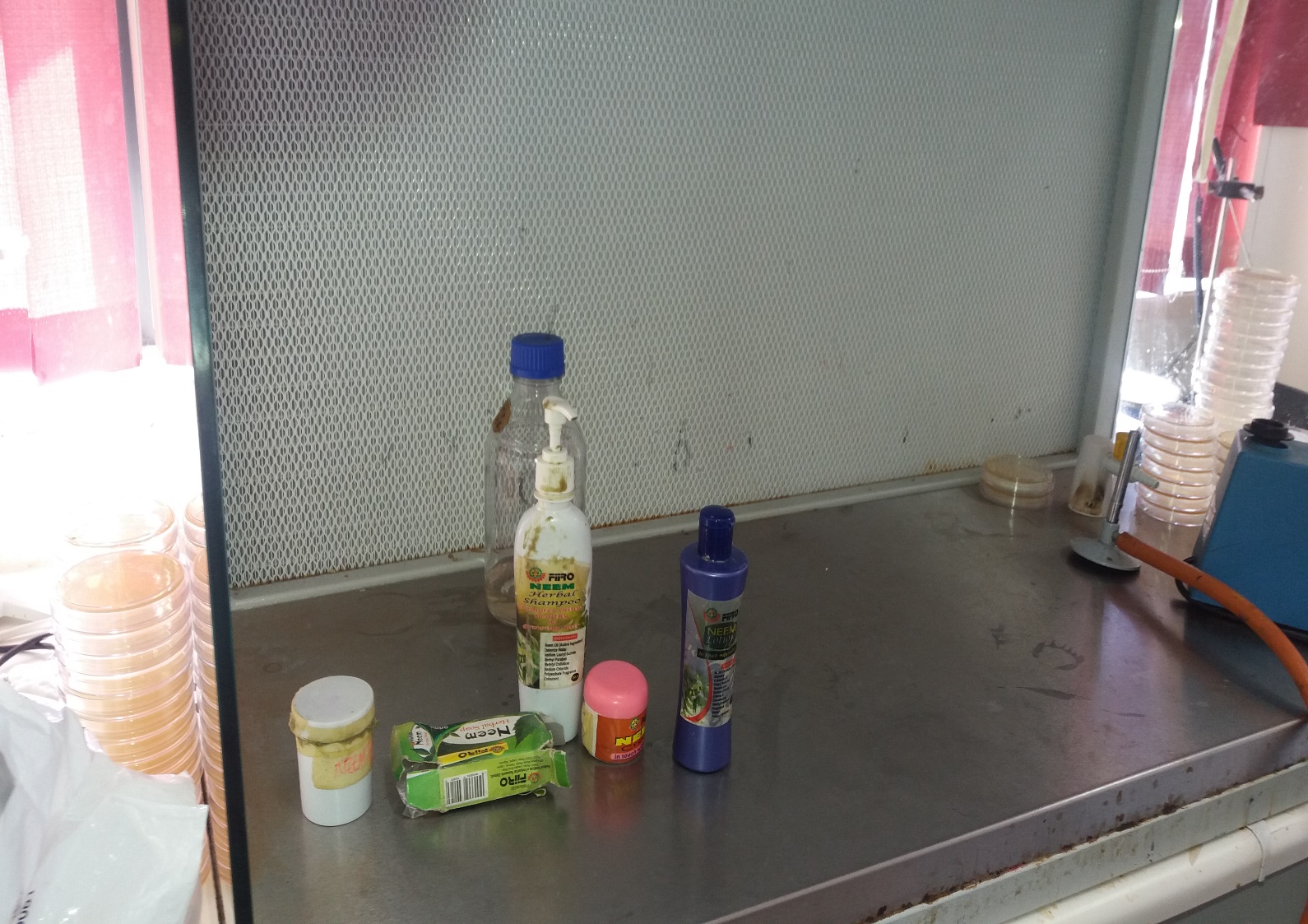
The Five neem products in laminar flow cabinet,** from the left toothpaste, soap, shampoo, cream and lotion)

**
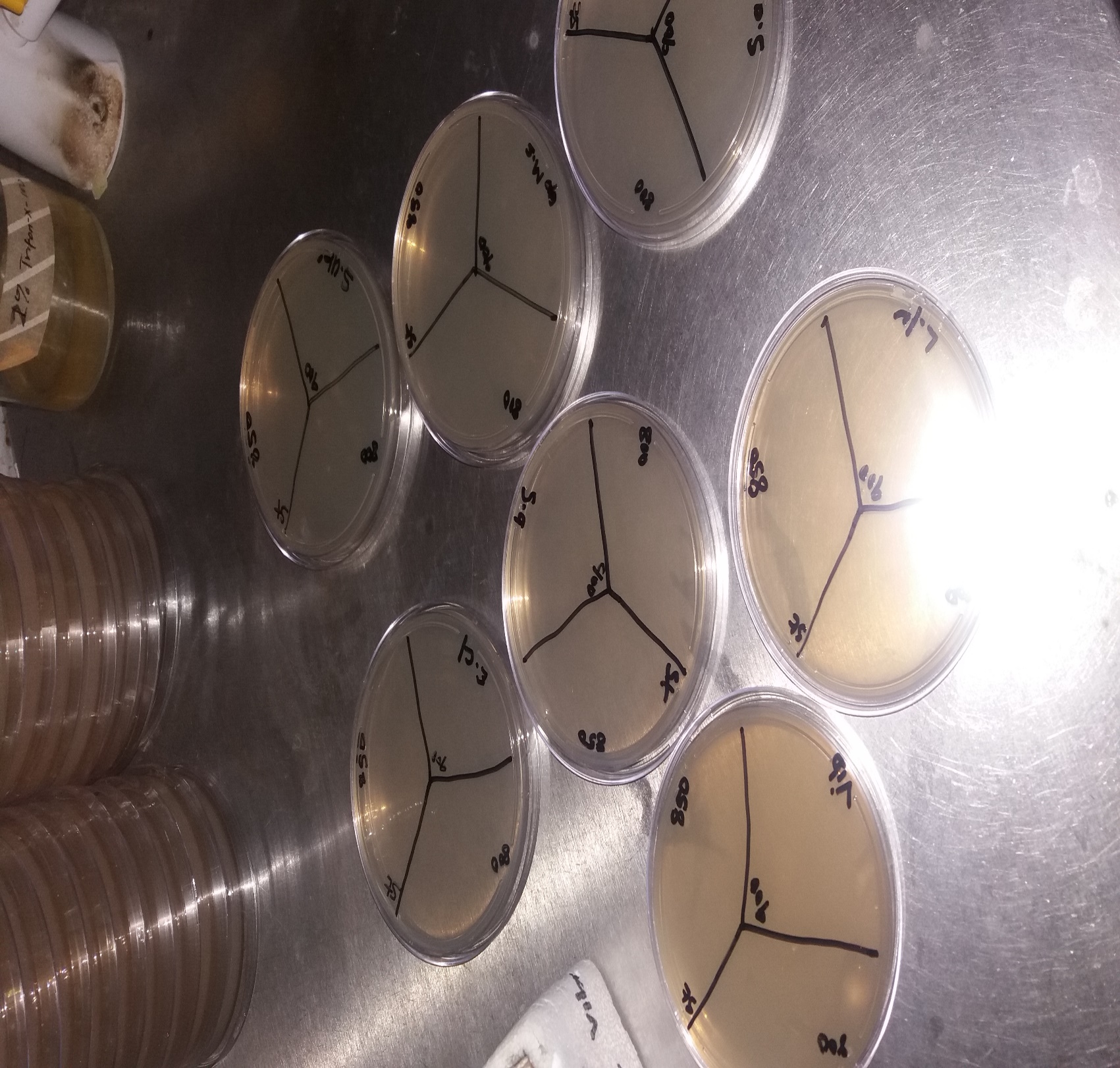
**

Screening of the products against 7 Bacterial strains at different concentrations

**
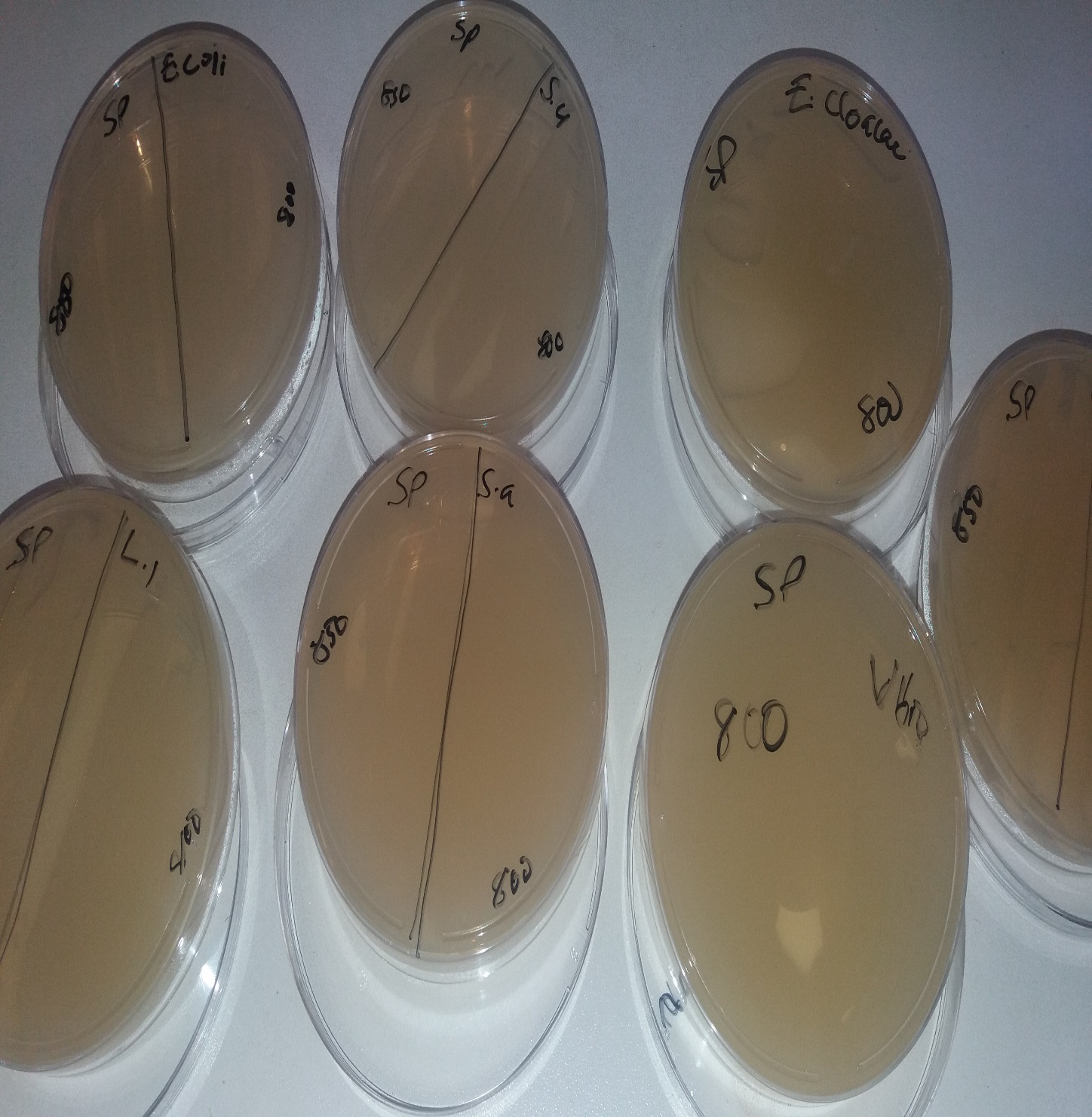
**

The neem soap (SP) screened against bacteria pathogens

**
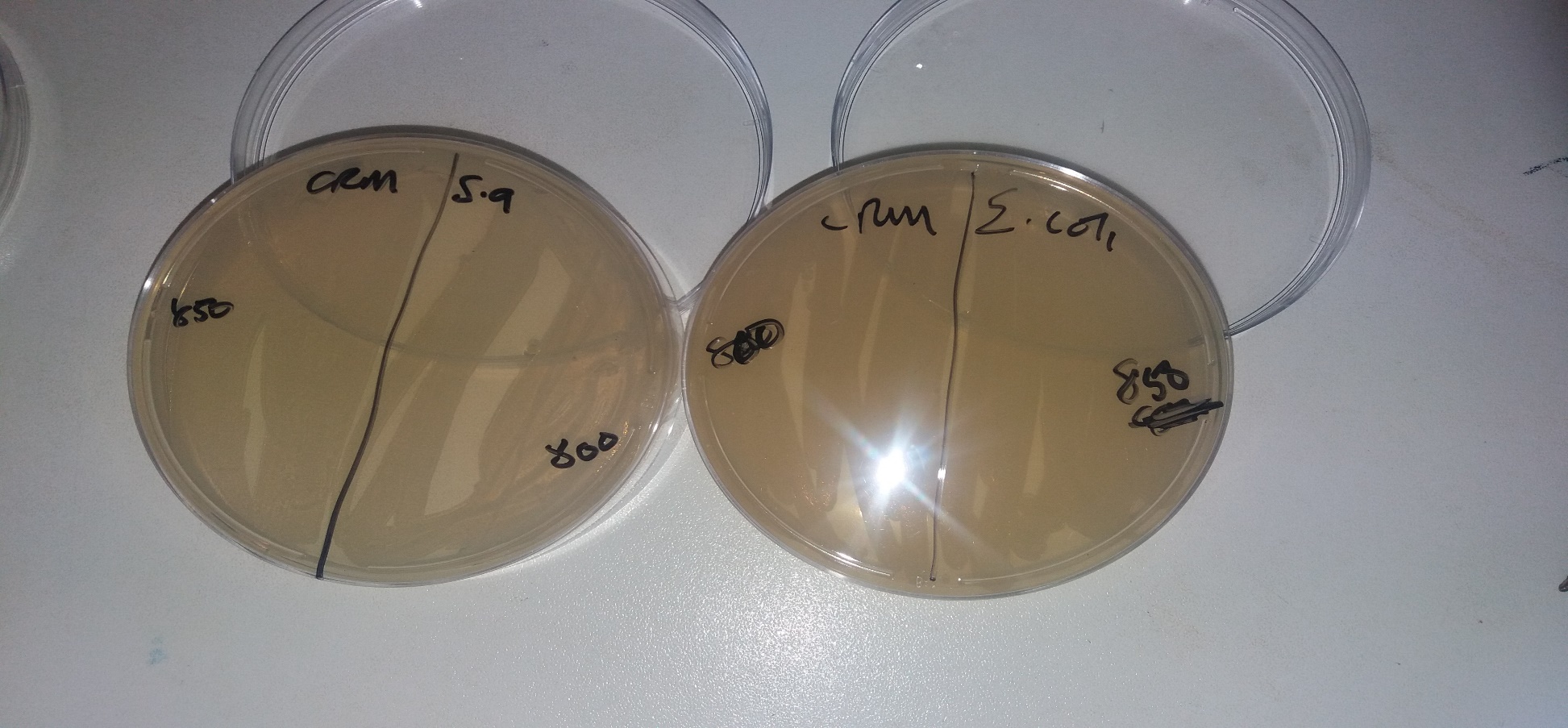
**

The neem cream (CRM) screened against *S. aureus* (Sa) and *E. coli* were bacteriostatic at 0.20 mg/mL.

***
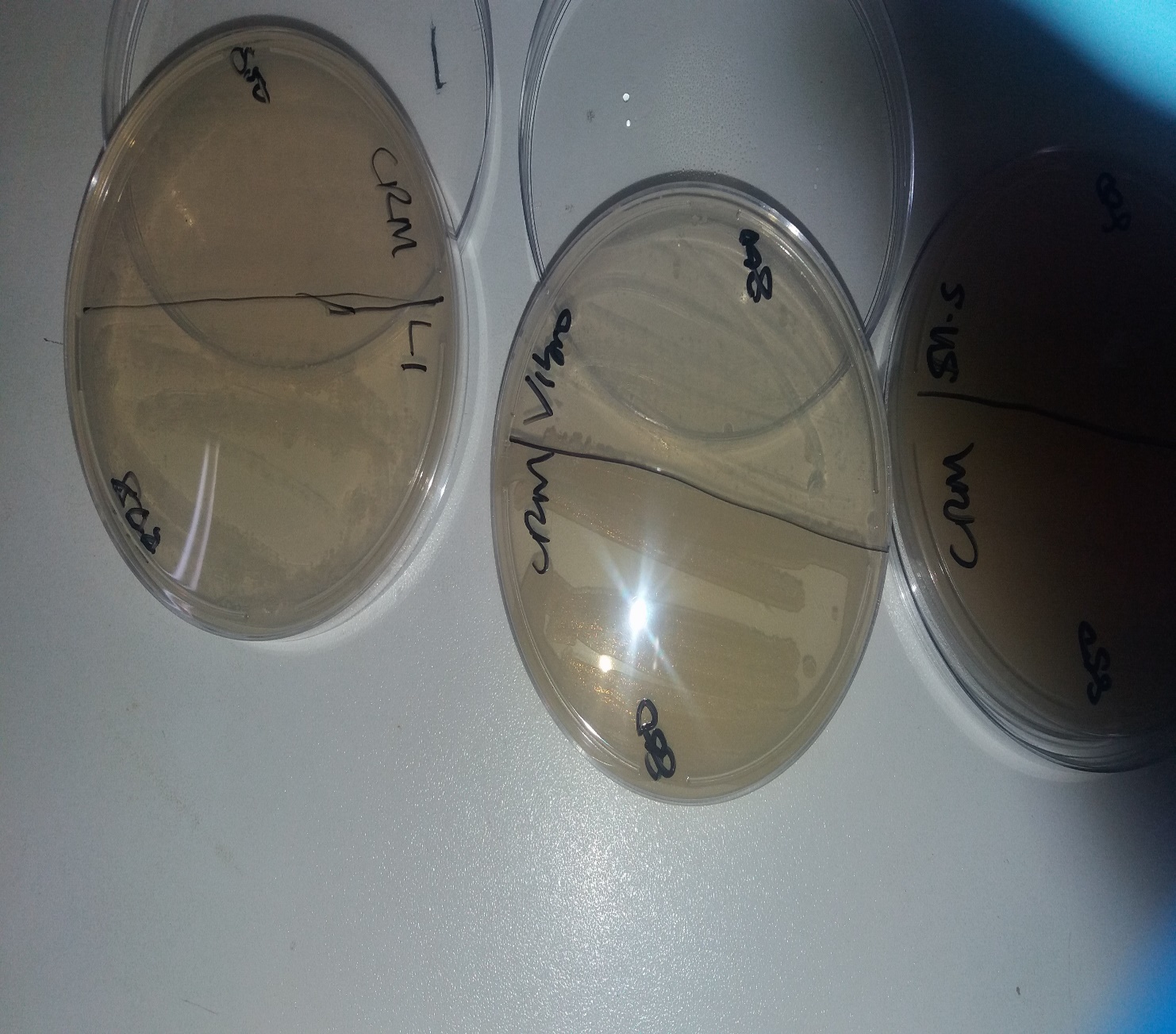
***

The neem products screened against *Listeria ivanovii* and *(LI)* and *Vibro* spp. were bacteriostatic and bactericidal at 0.20 and 0.40 mg/mL

***
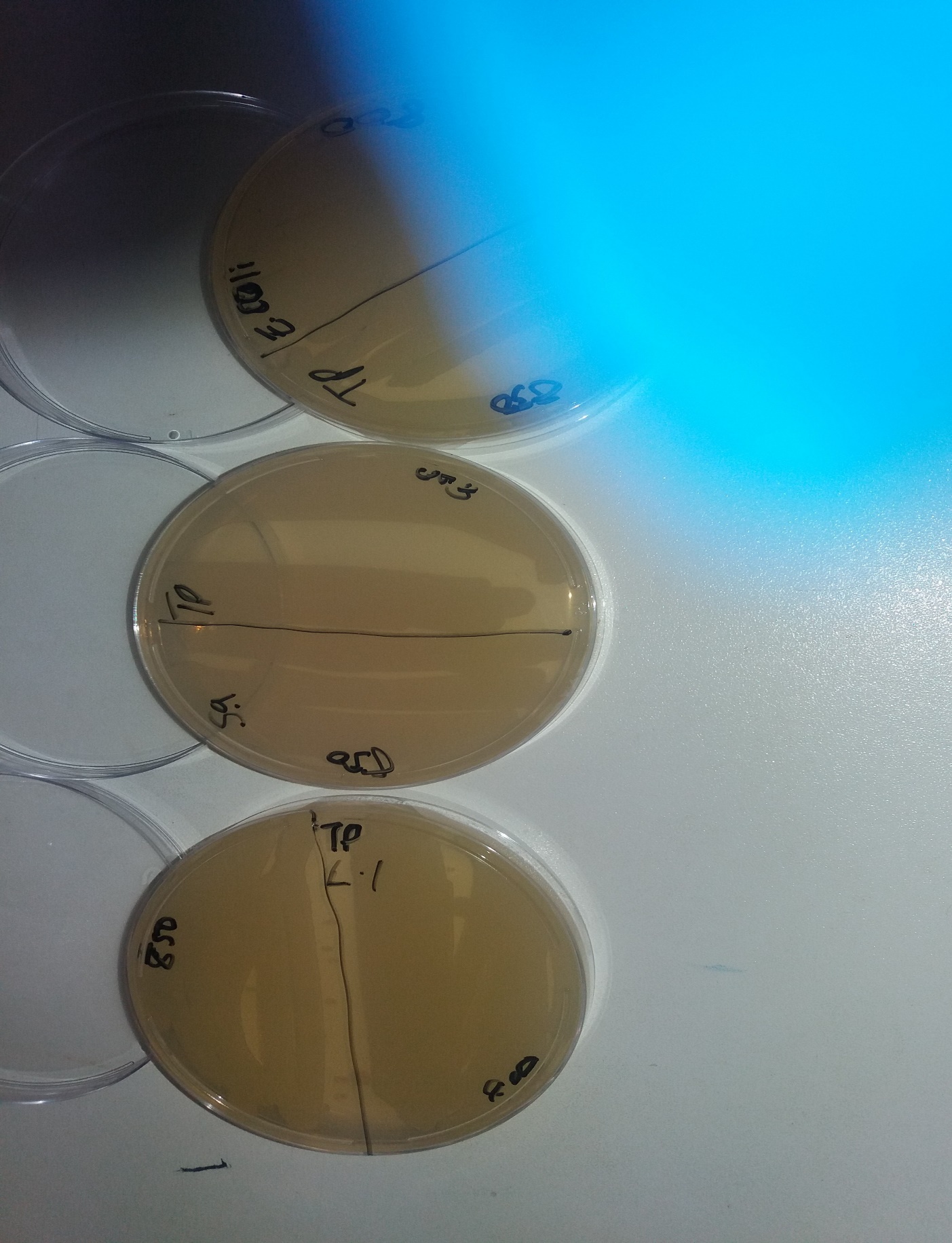
***

The neem toothpaste (TP) screened against *Listeria ivanovii* (LI), *S. aureus* (Sa) and *E. Coli*

**
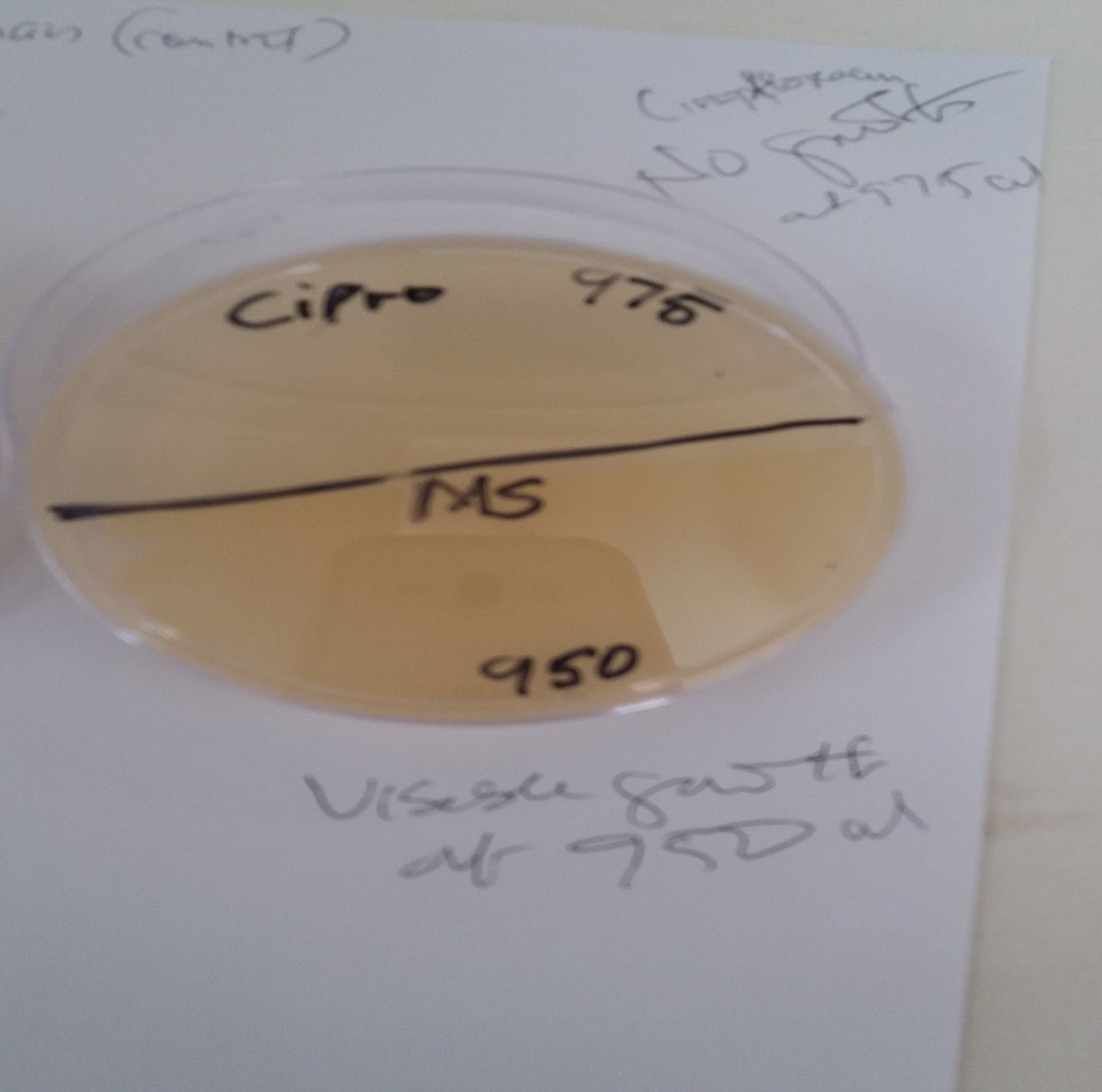
**

Ciprofloxacin (positive control), was bacteriostatic and bactericidal against *Mycobacterium. Smegmatis* and most of the test bacteria**.**

**
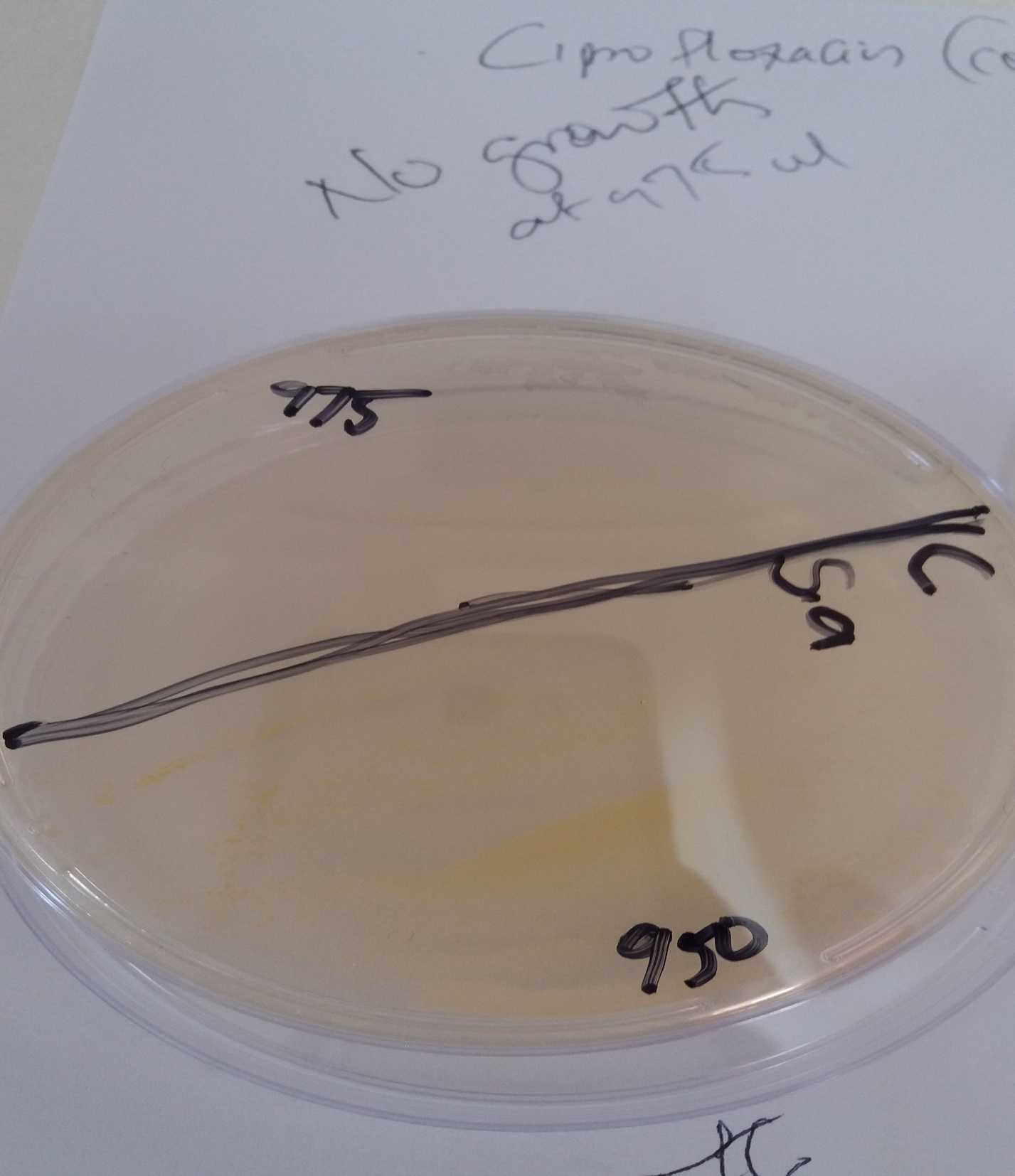
**

Ciprofloxacin (C) was bacteriostatic and bactericidal against *Staphylococcus aureus***.**
